# Efficacy and Safety of Bushenjiangya-Optimized Granule for Left Ventricular Diastolic Dysfunction in Hypertensive Patients: A Double-Blind, Randomized, Placebo-Controlled Trial

**DOI:** 10.1155/2020/7190352

**Published:** 2020-05-18

**Authors:** Qun Gao, Xiao-yun Cui, Fei Dong, Wen-ying Fan, Pin-hui Li, Jing Liu, Jin-jin Lu, Yan Meng, Wen-bai Qu, Kun Zhou, Qian Lin

**Affiliations:** ^1^Department of Cardiology, Dongzhimen Hospital, Beijing University of Chinese Medicine, Beijing 100070, China; ^2^Department of Cardiology, Dongfang Hospital, Beijing University of Chinese Medicine, Beijing 100078, China; ^3^Beijing University of Chinese Medicine, Beijing 100029, China; ^4^Department of Acupuncture, Qinghai Hospital of Traditional Chinese Medicine, Xining 810000, China; ^5^Department of Cardiology, Hainan Provincial Hospital of Traditional Chinese Medicine, Haikou 570203, China; ^6^Scientific Research Division, Dongfang Hospital, Beijing University of Chinese Medicine, Beijing 100078, China

## Abstract

**Objective:**

The study aimed to evaluate the efficacy and safety of Bushenjiangya-optimized (BSJYO) granule on left ventricular diastolic dysfunction (LVDD) in hypertensive (HTN) patients.

**Methods:**

120 patients diagnosed with HTN plus LVDD were randomly assigned to the BSJYO granule group and placebo group, and all patients received basal western medicine (WM) treatment. After eight weeks of treatment, we evaluated echocardiography, traditional Chinese medicine (TCM) syndromes, 24-hour ambulatory blood pressure, liver and kidney functions, and adverse events. Major adverse cardiovascular events (MACEs) were collected at 6-month follow-up.

**Results:**

Compared with pretreatment, E/Ea (Doppler-derived index of filling pressure and worsening LVDD) significantly decreased significantly after 8 weeks of treatment in the BSJYO granule plus basal WM group (10.52 ± 1.87 vs. 9.49 ± 1.49, *P* < 0.01), alongside reductions in significantly effective response (SER), effective response (ER), and total effective response (TER = SER + ER) in TCM symptom scores (21.59% vs. 71.70%, *P* < 0.01). There were no differences between treatment groups in kidney and liver function, early adverse events, or MACE.

**Conclusion:**

BSJYO granule plus basal WM is an effective and safe therapy for HTN patients with LVDD.

## 1. Introduction

Left ventricular diastolic dysfunction (LVDD) is characterized by abnormalities in left ventricular filling, including decreased diastolic distensibility and impaired relaxation, and it may represent an important pathophysiologic link between hypertension (HTN) and heart failure (HF), particularly in patients who have heart failure with normal or preserved ejection fraction (HFpEF) [1]. In a cross-sectional survey of Olmsted County community, asymptomatic LVDD was present on echocardiography in 47.3% of HTN patients ≥ 45 years. HTN individuals were three times more likely to have diastolic dysfunction (DD) compared to those without HTN [2]. In a more recent prospective cohort study, a history of HTN was present in 76% of patients with DD. In this cohort, HTN was found to be an independent predictor of the transition from DD to symptomatic HF [3].

Epidemiological evidence suggested the success of possible interventions: a mortality benefit has been observed in those whose LVDD improved compared with those whose LVDD remained the same or worsened [4]. Given the true paucity of LVDD-specific treatment trials and that LVDD can progress to HFpEF, current treatments mainly focus on the LVDD of HFpEF. Many studies, such as the Heart Outcomes Prevention Evaluation (HOPE) study [5] and the Antihypertensive and Lipid-Lowering Treatment to Prevent Heart Attack Trial (ALLHAT) study [6], suggested that good HTN control may lead to regression of LVDD [7]. Therefore, lowering blood pressure is critical for LVDD management.

Practically traditional Chinese medicine (TCM) has gained ground with established theories and obvious efficacy in the area of HTN treatment. Our clinical experience found that Liuwei Dihuang Pill and Three Herbs Decoction (*Selfheal*, *Radix gentianae*, *Motherwort*, *Peony*, *Liquorice*) worked well when used to treat HTN patients. In our study, Bushenjiangya-optimized (BSJYO) granules were created on the basis of the combination of the two formulas and used for the treatment of HTN. The purpose of our study was to investigate the efficacy and safety of this prescription in HTN patients with LVDD.

## 2. Methods

### 2.1. Study Design

This study was designed as a randomized, double-blind, placebo-controlled study in HTN patients with LVDD. The study protocol was reviewed and approved by the Ethics Committee of Dongfang Hospital and was registered at http://www.chictr.org.cn/showproj.aspx?proj=4901. The study was conducted in accordance with the principles of *the Good Clinical Practice Guidelines* and *the Declaration of Helsinki*. All patients signed written informed consent.

The study included 8 weeks of treatment and 6 months follow-up. All patients underwent physical examinations, standard blood pressure measurements, and laboratory blood and urine tests (including blood cell profile, NAG, urine microalbumin, AST, ALT, ALP, GGT, TBIL, BUN, Cr, and Glu) before and after treatment. The eligible patients were then randomly assigned, via the computer-generated block randomization method, to receive either BSJYO granule (including *astragalus membranaceus*, *salvia miltiorrhiza*, *rehmannia*, etc.) plus basal WM (western medicine) or placebo plus basal WM twice a day for totally 8 weeks.

### 2.2. Participants

We enrolled 120 HTN outpatients or inpatients with LVDD from Dongfang hospital through May 2014 to May 2016. The included patients should be clinically diagnosed as HTN with LVDD (Ea/Aa < 1 and E/Ea > 8), and the diagnosis followed the Guidelines for the Prevention and Treatment of Hypertension in China (revision 2010) and the Guidelines for the Diagnosis and Treatment of Common Diseases in Internal Medicine. Before enrollment, all the patients had taken lifestyle intervention or standard anti-HTN drug treatment and their blood pressure had been under control for a month. In addition, TCM diagnosis should be Yin deficiency of the liver and kidney viscera plus Qi deficiency and blood stasis.

However, we excluded the patient under the listed conditions: (a) diagnosed as secondary or malignant HTN; (b) attacked by acute cardiovascular and cerebrovascular events within 6 months before enrollment; (c) accompanied with cardiomyopathy, pericardiopathy, valvular disease, serious arrhythmia, diabetes or poor blood glucose control, hyperthyroidism, malignant tumors, mental disorders, and severe liver, kidney, hematopoietic system diseases; (d) pregnant or lactating women; and (e) participating or participated in other drug trials within one month.

### 2.3. Outcome Measures

The primary outcome was cardiac diastolic function measured by echocardiography. Ultrasonic echocardiography evaluation index mainly includes the early left ventricular diastolic blood flow velocity (E), left ventricular late diastolic blood flow velocity (A), early diastolic mitral valve ring motion amplitude (Ea), mitral valve ring late diastolic motion amplitude (Aa), E/A, Ea/Aa, E/Ea, and the each chamber size of the heart, room wall thickness, and left ventricular ejection fraction (EF). The examiner and reporter of ultrasonic echocardiography are special people who are not related to the study. The secondary outcomes included changes in symptom scores and response rate. The symptoms included Qi deficiency signs, such as exhaustion, fatigue, tired of speech, and sweat; Yin deficiency of the liver and kidney viscera signs, such as dizziness, dry eyes, numbness, tinnitus, dysphoria in the chest, palms, and soles, tidal fever, night sweating, dry mouth, insomnia, and constipation; and blood stasis signs, such as pricking on specific points. The intensity of above symptoms was rated as none, mild, moderate, or severe, which corresponded to scores of 0, 1, 2, and 3 points. An experienced and blinded TCM doctor performed the symptoms assessments. According to the symptom score, the response rate of each group was defined as follows, when compared with baseline: (a) significantly effective response (SER): symptom scores decreased equally to or more than 70%; (b) effective response (ER): symptom scores decreased by 30–70%; (c) no effective response (NER): symptom scores decreased less than 30% or even increased. We used the total effective response (TER = SER + ER) as the final response rate.

### 2.4. Sample Size

According to previous similar studies, we set TER to at least 25% higher in BSJYO granule plus basal WM treatment than 35% TER in placebo plus basal WM treatment with a Type I error of *α* = 0.05 and Type II error of *β* = 0.2. As we take 20% drop rate into consideration, the total sample size was 120 (60 cases in each group).

### 2.5. Randomization and Blinding

Patients were randomly assigned to the BSJYO plus basal WM group and placebo plus basal WM group by blocked randomization way. First, we generated random allocation scheme by SPSS software. Then, the corresponding group information was sealed in one light-proof envelope labeled with the corresponding case number. Through the whole trial, the random allocation scheme was locked in a file cabinet easily accessible by a statistician, and the case number was the only identity for each patient. Each case number was uniquely matched with the tags on the drug packaging. BSJYO granule and placebo were very similar in packaging, appearance, and taste. The administrator, patients, laboratory technicians, and data analyzers were all blinded to the random allocation scheme, only if emergency happened.

### 2.6. Statistical Analysis

We carried out per-protocol (PP) analysis. Continuous data were presented as mean ± standard deviation (SD). For continuous data, the independent *t*-test was used to do comparisons between groups, while the paired *t*-test for comparison within groups. For counting data, we used the chi-square test. All statistical analysis was performed by SPSS 21.0 software. A two-sided *P* value less than 0.05 was considered as statistically significant difference.

## 3. Results

**Figure 1:**
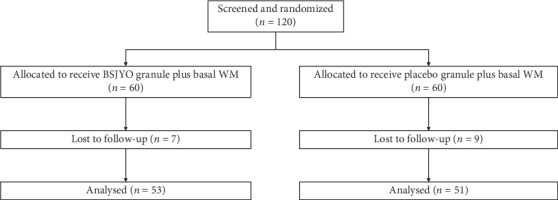
Participant flow chart.

From May 2014 to May 2016, 120 patients aged 18 to 80 years were enrolled in this study and were randomly assigned to receive BSJYO granule plus basal WM or the placebo plus basal WM treatment. The baseline characteristics of the patients are shown in Table 1. The two groups were comparable with respect to demographic characteristics, blood pressure, blood pressure load, and indexes of echocardiography. 16 out of 120 were excluded from data analysis because of failing to follow up or finishing the treatment (Figure 1).

After 8 weeks of treatment, a lower E/Ea was seen in the BSJYO granule plus basal WM group when compared with baseline, whereas the placebo plus basal WM group did not show; between groups, the BSJYO granule plus basal WM group demonstrated a significant lower E/Ea than the control group (shown in the Table 2).

In terms of the secondary endpoints, at baseline, there were no significant differences in TCM syndrome score between groups. Nevertheless, a significant lower score in the BSJYO granule plus basal WM group was observed when compared with the placebo group after 8 weeks of treatment (shown in Table 3). The BSJYO granule plus basal WM group showed a higher TER than the placebo group (shown in Table 4).

There was no difference between the two groups in terms of 24-hour ambulatory blood pressure (Table 5).

There were no MACEs, including coronary heart disease, malignant arrhythmia, heart failure, cardiogenic shock, and death, during the treatment nor at 6-month follow-up in the two groups. No differences in blood cell profile or liver and kidney function parameters were observed in either treatment arms over the course of the 8-week study (Table 6).

## 4. Discussion

Hypertension is a common cardiovascular and cerebrovascular disease, which is easily associated with damage to target organs such as the heart, brain, and kidney. Studies have confirmed that hypertension can cause changes in the structure and function of the myocardium, resulting in abnormal diastolic function and finally diastolic heart failure in patients. At present, most scholars believe that hypertension is the most common cause of diastolic dysfunction. Different from systolic heart failure, diastolic heart failure has not been effectively treated so far, and there is a lack of information to guide treatment. Therefore, in recent years, the focus of treatment for diastolic heart failure has entered the stage of comprehensive prevention and treatment focusing on controlling risk factors.

According to statistics, the incidence of diastolic dysfunction is on the rise. It also has higher hospitalization rates than systolic heart failure. Diastolic heart failure also decrease life quality of patients and spent a lot of medical resources, which brings heavy economic burden for the society. So, it is important to prevent and control it. Therefore, early diagnosis and treatment of LVDD are critical for stopping cardiac disease progress and benefiting their prognosis. Reversion of LVDD can not only avoid irreversible myocardial remolding but also greatly reserve systolic function.

TCM believes that Qi deficiency and blood stasis are the basic pathogenesis of chronic heart failure, which runs through the development of cardiac hypertrophy, diastolic heart dysfunction, systolic heart dysfunction, and pump failure. Deficiency of Qi and blood stasis and deficiency of both Yin and Yang are the pathologic changes of hypertensive left ventricular diastolic dysfunction and heart failure. Therefore, the early use of Qi-invigorating and blood-activating traditional Chinese medicine intervention caused by Qi deficiency an blood stasis,can prevent the further derivation of pathological symptoms, it is possible to improve the clinical symptoms of hypertension caused by left ventricular diastolic dysfunction and thereby delay or prevent its development, or even reverse it. In our study, we found that compared with the WM group, the symptoms of Qi deficiency, blood stasis, and liver and kidney deficiency in the BSJYO group were significantly improved.

However, comparing to the great progress of systolic heart failure treatment, persuasive treatments for diastolic heart failure are still lacking. Our previous research has found that tonifying Qi and activating blood herbs (such as *Astragalus*, *Codonopsis*, *Sanqi* and *Salvia*) can improve cardiomyocyte diastolic function and relieve calcium overload in the artery constriction-induced HF rat model, which is mainly through regulating the activity of calcium homeostasis-related proteins, including CaMKII [8]. Based on these discoveries and clinical experiences, we designed this study to testify the effect and safety of BSJYO granule plus basal WM on HTN with LVDD. According to the consensus announced by ESC in 2007, ratio between early mitral inflow velocity and mitral annular early diastolic velocity (E/Ea) is central in the guidelines for diastolic evaluation. In our study, E/Ea of the BSJYO granule plus WM group significantly decreased when compared with the baseline and placebo plus WM group, which indicated the benefit of BSJYO plus WM on LVDD. In addition, BSJYO granule plus WM also showed a lower symptom score when compared with the placebo plus WM group after treatment. However, because blood pressure of patients should be under control before enrollment, we did not find obvious blood pressure decrease in any group after treatment, and no MACEs happened during 6 months follow-up.

Based on the above evidence, BSJYO granule plus WM may be an effective and safe treatment for HTN with LVDD, but more robust conclusion should be established on rigorously designed RCT of multicenter, big size population, and long-term follow-up. To our best knowledge, our study is the first randomized, double-blind, placebo-controlled trial regarding TCM treatment for LVDD, which may provide clue for a promising way to treat LVDD.

The limitation of our study is that BMI was not taken as an observational indicator, which needs to be improved in further studies.

## 5. Conclusion

Compared with placebo plus WM, BSJYO granule plus WM can be safely and effectively used to treat HTN with LVDD at least 8 weeks.

## Figures and Tables

**Table 1:** Baseline data.

Item	Category	Placebo + WM group (*n* = 60)	BSJYO granule + WM group (*n* = 60)
Gender	Male	31 (51.7%)	29 (48.3%)
	Female	32 (53.3%)	28 (46.6%)
Age (year)		61.77 ± 10.65	60.28 ± 9.20
Blood pressure (mmHg)	SBP	130.05 ± 8.89	130.21 ± 7.62
	DBP	78.72 ± 7.86	78.54 ± 8.51
	MAP	94.08 ± 9.51	94.28 ± 7.71
	HR	70.03 ± 6.74	68.56 ± 8.99
	PP	54.05 ± 9.34	52.54 ± 8.06
Blood pressure load (%)	Daytime SBP	34.91 ± 15.68	34.95 ± 14.66
	Daytime DBP	33.06 ± 15.64	32.74 ± 21.27
	Night SBP	38.94 ± 21.14	41.95 ± 26.89
	Night SBP	39.83 ± 16.04	39.74 ± 31.41
Echocardiography	LVEF	63.54 ± 6.04	63.74 ± 6.03
	E/Ea	10.24 ± 2.49	10.53 ± 2.48
	Ea/Aa	0.65 ± 0.16	0.66 ± 0.12

*Note*. SBP = systolic blood pressure; DBP = diastolic blood pressure; MAP = mean arterial pressure; HR = heart rate; PP = pulse pressure; LVEF = left ventricular ejection fraction.

**Table 2:** E/Ea comparison (mean ± SD).

Items		Placebo + WM group (*n* = 51)		BSJYO granule + WM group (*n* = 53)	
		Baseline	8^th^ week	Baseline	8^th^ week
LA		33.83 ± 5.01	34.83 ± 4.67	33.49 ± 3.60	32.74 ± 3.65
LVIDs		31.80 ± 5.93	31.60 ± 6.19	32.36 ± 6.94	32.31 ± 5.88
LVIDd		46.43 ± 7.00	45.34 ± 8.77	47.51 ± 5.49	45.85 ± 4.87
LVSTd		8.91 ± 1.82	9.03 ± 1.99	8.92 ± 1.20	8.67 ± 0.90
LVSTs		12.34 ± 2.21	12.54 ± 2.06	11.85 ± 1.31	11.87 ± 1.20
LVPWd		9.49 ± 1.60	9.00 ± 1.41	9.64 ± 1.91	9.23 ± 1.22
LVPWs		12.31 ± 1.68	12.46 ± 1.67	12.79 ± 2.09	12.74 ± 1.92
EF		63.57 ± 6.04	64.66 ± 6.85	63.74 ± 6.03	64.36 ± 4.98
E		0.72 ± 0.15	0.70 ± 0.13	0.72 ± 0.16	0.74 ± 0.14
A		0.83 ± 0.18	0.82 ± 0.15	0.81 ± 0.13	0.82 ± 0.14
Ea		0.07 ± 0.02	0.07 ± 0.02	0.07 ± 0.02	0.09 ± 0.08
Aa		0.14 ± 0.13	0.14 ± 0.13	0.11 ± 0.02	0.11 ± 0.02
E/A		0.91 ± 0.27	0.87 ± 0.18	0.90 ± 0.24	0.94 ± 0.28
Ea/Aa		0.70 ± 0.20	0.64 ± 0.16	0.68 ± 0.15	0.71 ± 0.15
E/Ea		9.93 ± 2.43	9.84 ± 2.42	10.52 ± 1.87	9.49 ± 1.49^*∗*#^

*Note*. BSJYO = Bushenjiangya-optimized granule. *∗P* < 0.01 compared with the placebo group; ^#^*P* < 0.01 compared with baseline.

**Table 3:** TCM syndrome score (mean ± SD).

Syndrome	Symptoms	Placebo + WM group (*n* = 51)		BSJYO granule + WM group (*n* = 53)	
		Baseline	8^th^ week	Baseline	8^th^ week
Qi deficiency	Exhaustion	1.14 ± 0.45	0.96 ± 0.60	1.28 ± 0.57	0.70 ± 0.61^##*∗∗*^
	Fatigue	1.31 ± 0.47	1.14 ± 0.63	1.51 ± 0.64	0.64 ± 0.65^##*∗∗*^
	Tired of speech	0.84 ± 0.61	0.73 ± 0.57	0.85 ± 0.82	0.43 ± 0.57^##*∗*^
	Sweat	0.90 ± 0.61	0.76 ± 0.55	0.96 ± 0.62	0.75 ± 0.70
Liver and kidney Yin deficiency	Dizziness	1.20 ± 0.57	1.00 ± 0.35^#^	1.08 ± 0.55	0.66 ± 0.55^##^
	Dry eyes	1.04 ± 0.45	0.96 ± 0.34	1.19 ± 0.79	0.66 ± 0.62^##*∗*^
	Numbness	0.84 ± 0.46	0.76 ± 0.47	0.87 ± 0.52	0.58 ± 0.57^##^
	Tinnitus	0.98 ± 0.88	0.76 ± 0.79	0.96 ± 0.85	0.58 ± 0.72^##^
	Dysphoria in chestpalms soles	0.59 ± 0.64	0.59 ± 0.50	0.74 ± 0.52	0.43 ± 0.50^##*∗*^
	Tidal fever and night sweating	0.88 ± 0.71	0.67 ± 0.59	1.04 ± 0.59	0.38 ± 0.49^##*∗∗*^
	Dry mouth	1.16 ± 0.64	1.00 ± 0.75	1.32 ± 0.83	0.92 ± 0.62^##^
	Insomnia	1.14 ± 0.53	0.90 ± 0.50^#^	1.04 ± 0.55	0.47 ± 0.61^##*∗*^
	Constipation	1.12 ± 0.86	0.88 ± 0.62	0.96 ± 0.76	0.45 ± 0.58^##^
Blood stasis	Pricking on specific points	1.06 ± 0.42	1.00 ± 0.20	1.09 ± 0.49	0.21 ± 0.41^##*∗∗*^

^*∗*^ *P* < 0.05 compared with the placebo group; ^*∗∗*^*P* < 0.01 compared with the placebo group. ^#^*P* < 0.05 compared with baseline; ^##^*P* < 0.01 compared with baseline.

**Table 4:** Total effective response (mean ± SD).

Groups	*N*	SER	ER	NER	TER (%)
Placebo + WM	51	2 (3.90%)	9 (17.56%)	40 (78.41%)	21.59
BSJYO granule + WM	53	7 (13.21)^*∗*^	31 (58.59%)^*∗*^	15 (28.30%)^*∗*^	71.70^*∗*^

*Note*. SER = significantly effective response; ER = effective response; NER = no effective response; TER = total effective response. ^*∗*^*P* < 0.05 compared with the placebo group.

**Table 5:** 24-hour ambulatory blood pressure.

Items	Placebo + WM		BSJYO granule + WM	
	Baseline	8^th^ week	Baseline	8^th^ week
Daytime SBP	34.91 ± 15.68	35.03 ± 16.06	34.95 ± 14.66	31.64 ± 16.25
Daytime DBP	33.06 ± 15.64	32.63 ± 16.50	32.74 ± 21.27	33.26 ± 20.31
Night SBP	38.94 ± 21.14	38.34 ± 18.50	41.95 ± 26.99	39.72 ± 28.46
Night DBP	39.83 ± 16.04	39.51 ± 16.15	39.74 ± 31.41	39.26 ± 24.00

**Table 6:** Safety outcome (mean ± SD).

Indexes		Placebo + WM group		BSJYO granule + WM group	
		Baseline	8^th^ week	Baseline	8^th^ week
Blood cell profile	WBC (^*∗*^10^9^)	6.75 ± 2.13	6.54 ± 1.27	6.601 ± 2.07	6.14 ± 1.35
	RBC (^*∗*^10^9^)	4.54 ± 0.44	4.73 ± 0.50	4.68 ± 0.51	4.74 ± 0.53
	HGB (g/l)	141.0 ± 16.60	139.64 ± 14.39	141.56 ± 14.36	136.88 ± 13.56
	PLT (^*∗*^10^9^)	210.30 ± 43.66	209.39 ± 37.52	232.25 ± 58.70	229.09 ± 56.79
Liver and kidney function	ALT (U/l)	25.8 ± 18.66	26.32 ± 15.85	24.5 ± 11.45	23.33 ± 8.74
	AST (U/l)	22.9 ± 10.17	23.37 ± 8.60	24.30 ± 9.08	24.41 ± 7.37
	ALP (U/l)	76.2 ± 22.65	74.04 ± 22.22	83.2 ± 23.78	81.12 ± 25.19
	GGT (U/l)	28.3 ± 18.68	30.81 ± 21.14	23.9 ± 14.05	24.88 ± 13.80
	TBIL (*μ*mol)	12.90 ± 4.07	13.76 ± 4.28	13.14 ± 5.59	12.86 ± 6.17
	BUN (mmol)	5.71 ± 1.56	6.10 ± 1.31	5.19 ± 1.10	5.19 ± 1.21
	CR (*μ*mol)	71.7 ± 18.90	72.93 ± 17.03	66.6 ± 15.62	61.72 ± 21.29
	GLU (mmol)	6.22 ± 1.84	6.27 ± 1.63	6.39 ± 1.91	5.82 ± 1.31
Early kidney damage	MALB (mg/l)	12.74 ± 5.39	13.15 ± 6.85	12.22 ± 7.92	11.40 ± 10.76
	NAG (IU/l)	8.55 ± 3.15	8.54 ± 3.37	8.18 ± 6.25	8.40 ± 6.59

*Note*. WBC = white blood cell; RBC = red blood cell; HGB = hemoglobin; PLT = platelet; ALT = alanine transaminase; AST = glutamic-oxaloacetic transaminase; ALP = alkaline phosphatase; GGT = gamma-glutamyl transpeptidase; TBIL = total bilirubin; BUN = blood urea nitrogen; CR = creatinine; GLU = glucose; MALB = microalbuminuria; NAG = glucosidase.

## Data Availability

The data used to support the findings of this study are included within the article.
